# Supplementary material for: Building systems for preparedness: Global scoping studies on institutional governance and National Public Health Agencies
Source: PLOS Glob Public Health. 2026 Feb 12;6(2):e0005427. doi: 10.1371/journal.pgph.0005427 (PMC12900297; doi:10.1371/journal.pgph.0005427)
Supplement: S1 Table — This table presents the full electronic search strategies used across PubMed, Scopus, Web of Science, EMBASE, CINAHL, Cochrane Library, and Google Scholar. (DOCX) [file pgph.0005427.s002.docx]

S1 Table. Search Strategy Across Databases

| **Database** | **Search Terms / Strategy** | **Identified Articles** |
| --- | --- | --- |
| **PubMed** | (("Disaster Planning"[MeSH Terms] OR "Public Health Emergency"[Title/Abstract] OR "public health emergency management"[Title/Abstract] OR "health emergency preparedness"[Title/Abstract] OR "emergency response"[Title/Abstract]) AND ("Public Health Administration"[MeSH Terms] OR "National Public Health Institute"[Title/Abstract] OR "public health institute"[Title/Abstract] OR "national public health agency"[Title/Abstract] OR "NPHI"[Title/Abstract] OR "national institute of public health"[Title/Abstract] OR "public health agency"[Title/Abstract]) AND ("Governance"[Title/Abstract] OR "Autonomy"[Title/Abstract] OR "institutional arrangements"[Title/Abstract] OR "Preparedness"[Title/Abstract] OR "Response Functions"[Title/Abstract] OR "Surveillance"[Title/Abstract] OR "workforce"[Title/Abstract] OR "capacity building"[Title/Abstract] OR "training"[Title/Abstract] OR "Field Epidemiology Training Program"[Title/Abstract] OR "financing"[Title/Abstract] OR "funding"[Title/Abstract] OR "subnational"[Title/Abstract] OR "regional"[Title/Abstract] OR "coordination"[Title/Abstract] OR "community engagement"[Title/Abstract])) AND ((ffrft[Filter]) AND (humans[Filter]) AND (english[Filter])) | 200 |
| **Scopus** | TITLE-ABS-KEY(("National Public Health Institute" OR "public health emergency" OR "emergency preparedness" OR "EPR") AND ("governance" OR "institutional arrangements" OR "workforce development" OR "financing" OR "preparedness functions"))AND (LIMIT-TO (LANGUAGE, "English")) | 815 |
| **Web of Science** | TS=("National Public Health Institute" OR "public health emergency management" OR "emergency preparedness") AND TS=("governance" OR "response functions" OR "financing" OR "workforce" OR "capacity"). English | 961 |
| **EMBASE** | ('public health emergency'/exp OR 'emergency preparedness' OR 'EPR') AND ('National Public Health Institute' OR 'institutional governance' OR 'workforce' OR 'capacity' OR 'financing'). Emtree + free-text; English, 2015–2025. | 950 |
| **CINAHL** | (TI ("National Public Health Institute" OR "public health institute"  OR "public health agency" OR NPHI)  OR AB ("National Public Health Institute" OR "public health institute"  OR "public health agency" OR NPHI)) AND (TI ("emergency preparedness" OR "public health emergency management"  OR "emergency response" OR "EPR") OR AB ("emergency preparedness" OR "public health emergency management"  OR "emergency response" OR "EPR")) AND (TI ("governance" OR "autonomy" OR "institutional arrangements"  OR "preparedness functions" OR "surveillance" OR "incident management" OR "PHEOC"  OR "workforce development" OR "capacity building" OR "training" OR "surge capacity"  OR "financing" OR "funding" OR "subnational" OR "regional"  OR "community engagement" OR "coordination")  OR AB ("governance" OR "autonomy" OR "institutional arrangements"  OR "preparedness functions" OR "surveillance" OR "incident management" OR "PHEOC"  OR "workforce development" OR "capacity building" OR "training" OR "surge capacity"  OR "financing" OR "funding" OR "subnational" OR "regional"  OR "community engagement" OR "coordination")) AND (LA English)  AND (DT 20150101-20251231) | 1,043 |
| **Cochrane Library** | ("National Public Health Institute" OR "public health institute"  OR "public health agency" OR NPHI) AND ("emergency preparedness" OR "public health emergency management"  OR "emergency response" OR "EPR”) AND ("governance" OR "autonomy" OR "institutional arrangements"  OR "preparedness functions" OR "surveillance" OR "incident management" OR "PHEOC” OR "workforce development" OR "capacity building" OR "training" OR" surge capacity” OR "financing" OR "funding" OR "subnational" OR "regional"  OR "community engagement" OR "coordination") :ti,ab,kw | 5 |
| **Google Scholar** | intitle:("National Public Health Institute" OR "public health agency") AND ("emergency preparedness" OR "public health emergency management") AND ("governance" OR "autonomy") AND ("preparedness functions" OR "surveillance" OR "incident management" OR "PHEOC") AND ("workforce development" OR "training" OR "surge capacity") AND ("financing" OR "sustainability") AND ("subnational" OR "regional offices") AND ("community engagement" OR "coordination") | 189 |
