# Supplementary material for: Building systems for preparedness: Global scoping studies on institutional governance and National Public Health Agencies
Source: PLOS Glob Public Health. 2026 Feb 12;6(2):e0005427. doi: 10.1371/journal.pgph.0005427 (PMC12900297; doi:10.1371/journal.pgph.0005427)
Supplement: S2 Table — This table lists all studies assessed at full-text stage (n = 98), indicating included and excluded articles with specific reasons for exclusion. (DOCX) [file pgph.0005427.s003.docx]

S1 Table. Inventory of all full-text assessed studies (n = 98), showing included (n = 60) and excluded (n = 38) articles with specific reasons for exclusion.

| **ID** | **Authors** | **Year** | **Region** | **Type** | **Decision** | **Specific Reason** |
| --- | --- | --- | --- | --- | --- | --- |
| 1 | Ostermann et al. | 2023 | Global | Policy analysis | Included |  |
| 2 | Frieden & Koplan | 2010 | Global | Policy commentary | Included |  |
| 3 | Herstein et al. | 2021 | Global | Review | Included |  |
| 4 | El-Jardali et al. | 2025 | Global | Tool validation | Excluded | Focused on development and initial validation of an assessment tool rather than empirical analysis of national PHEPR systems. |
| 5 | Myhre et al. | 2022 | Global | Scoping review | Included |  |
| 6 | Sasie et al. | 2024 | Ethiopia | Empirical | Included |  |
| 7 | Abou-Taleb et al. | 2024 | EMR | Empirical | Included |  |
| 8 | Vernaccini et al. | 2025 | Global | Metric study | Excluded | Presented a methodological preparedness metric without analysis of institutional governance or system performance. |
| 9 | Khatri et al. | 2023 | Global | Qualitative synthesis | Included |  |
| 10 | Ryan et al. | 2023 | Global | Narrative review | Excluded | Conceptual discussion of resilience strategies without empirical assessment of preparedness institutions. |
| 11 | Utheim et al. | 2023 | Europe | Review | Excluded | Limited to simulation exercises and after-action reviews rather than national preparedness systems. |
| 12 | Chiossi et al. | 2021 | Global | Scoping review | Included |  |
| 13 | Calonge et al. | 2020 | Global | Policy report | Included |  |
| 14 | Sasie et al. | 2025 | Ethiopia | Validation study | Included |  |
| 15 | Savoia et al. | 2009 | Global | Review | Included |  |
| 16 | Príncipe et al. | 2023 | Global | Modelling review | Excluded | Focused on decision-support modelling and not on real-world public health emergency systems. |
| 17 | Lamberti-Castronuovo et al. | 2022 | Global | Review | Included |  |
| 18 | English et al. | 2024 | Global | Scoping review | Included |  |
| 19 | Kandel et al. | – | Global | Methods paper | Excluded | Focused on updating a reporting tool rather than system-level preparedness evaluation. |
| 20 | Yang et al. | 2022 | Global | Bibliometric study | Included |  |
| 21 | Rubinelli et al. | 2022 | Global | Framework | Included |  |
| 22 | Copper et al. | 2020 | Global | Descriptive analysis | Excluded | Analyzed outputs of exercises only; did not evaluate governance or institutional preparedness. |
| 23 | Haeberer et al. | 2020 | Global | Tool’s review | Excluded | Reviewed preparedness assessment tools rather than analyzing PHEPR systems. |
| 24 | Sasie et al. | 2025 | Ethiopia | Scoping review | Included |  |
| 25 | Karo et al. | 2018 | Bangladesh | Empirical | Included |  |
| 26 | Chen et al. | 2022 | Global | Empirical | Included |  |
| 27 | Khan et al. | 2019 | Global | Methods study | Excluded | Developed indicators without assessing national preparedness implementation. |
| 28 | Brito et al. | 2022 | Global | Empirical | Included |  |
| 29 | Gurley et al. | 2021 | Global | Policy analysis | Included |  |
| 30 | Hung et al. | 2022 | Global | Delphi study | Included |  |
| 31 | Foo et al. | 2022 | Asia | Comparative study | Included |  |
| 32 | Kandel et al. | 2020 | Global | Descriptive study | Excluded | Aggregated IHR reporting data without examination of institutional structures. |
| 33 | Derese et al. | 2025 | Ethiopia | Empirical | Included |  |
| 34 | El-Jardali et al. | – | MENA | Conceptual framework | Excluded | Theoretical framework without empirical system assessment. |
| 35 | Wright et al. | 2024 | Global | Review | Included |  |
| 36 | Meyer et al. | 2020 | Global | Framework | Included |  |
| 37 | Durski et al. | 2020 | Global | Policy analysis | Included |  |
| 38 | Vernaccini et al. | – | Global | Technical report | Excluded | Companion methodological document without independent systems analysis. |
| 39 | Zhang et al. | 2023 | Global | Review | Included |  |
| 40 | Haldane et al. | 2021 | Multi-country | Comparative | Included |  |
| 41 | Berkessa et al. | 2025 | Ethiopia | Empirical | Included |  |
| 42 | Zhang et al. | 2025 | China | Index development | Excluded | Created an urban resilience index outside national PHEPR scope. |
| 43 | Oppenheim et al. | 2019 | Global | Index study | Excluded | Constructed a global preparedness index without institutional analysis. |
| 44 | Oppenheim et al. | – | Global | Data mapping | Excluded | Open-source mapping exercise not evaluating preparedness systems. |
| 45 | Mackenzie et al. | 2014 | Global | Program analysis | Included |  |
| 46 | Cordes et al. | 2017 | Global | Empirical | Included |  |
| 47 | Kluge et al. | 2018 | Global | Policy analysis | Included |  |
| 48 | Vernaccini et al. | – | Global | Methods paper | Excluded | Integration of risk data without governance or systems assessment. |
| 49 | Erondu et al. | 2021 | Global | Methods paper | Excluded | Development of indicators rather than evaluation of preparedness institutions. |
| 50 | Nabatanzi et al. | 2023 | Uganda | Report | Excluded | Described national planning without analytic evaluation of systems. |
| 51 | Nuzzo et al. | 2019 | Global | Scoping review | Included |  |
| 52 | Stehling-Ariza et al. | 2017 | Global | Program evaluation | Included |  |
| 53 | Martinez et al. | 2019 | USA | Empirical | Included |  |
| 54 | Tahir et al. | 2025 | Pakistan | Empirical | Included |  |
| 55 | Wilbroda et al. | 2024 | Kenya | Empirical | Included |  |
| 56 | Czabanowska & Kuhlmann | 2021 | Global | Competency study | Excluded | Focused on competencies rather than PHEPR systems. |
| 57 | Moore et al. | 2025 | Global | Workforce study | Excluded | Practitioner competency framework without systems assessment. |
| 58 | Chambers et al. | 2025 | Global | Methods review | Excluded | Evaluated exercise methods rather than institutional preparedness. |
| 59 | Peterson et al. | 2024 | Global | Review | Excluded | Operational strategies discussed without analysis of governance structures. |
| 60 | Horney et al. | 2021 | USA | Empirical | Included |  |
| 61 | Lee et al. | 2023 | Global | Scoping review | Included |  |
| 62 | Murthy et al. | 2017 | USA | Empirical | Included |  |
| 63 | Marron et al. | 2025 | Ireland | Narrative review | Included |  |
| 64 | Ongesa et al. | 2025 | Global | Empirical | Included |  |
| 65 | Nelson et al. | 2007 | Global | Conceptual | Included |  |
| 66 | Stoto et al. | 2017 | Europe | Framework | Included |  |
| 67 | Chiang et al. | 2020 | USA | Framework | Included |  |
| 68 | Hunter et al. | 2023 | Global | Workforce study | Excluded | Education and training focus without system-level preparedness analysis. |
| 69 | Kennedy et al. | 2022 | USA | Empirical | Included |  |
| 70 | Bedi et al. | 2021 | India | Review | Included |  |
| 71 | Lee & Errett | – | Global | Review article | Excluded | Discussion of research priorities without empirical systems evaluation. |
| 72 | Fu et al. | 2021 | China | Empirical | Included |  |
| 73 | Davis et al. | 2021 | USA | Empirical | Included |  |
| 74 | Woldetsadik et al. | 2021 | Multi-country | Program evaluation | Excluded | Donor program assessment not focused on national PHEPR systems. |
| 75 | Hao et al. | 2024 | China | Empirical | Included |  |
| 76 | Khan et al. | 2018 | Global | Framework | Included |  |
| 77 | Hunter et al. | 2022 | Global | Workforce roadmap | Excluded | Workforce planning document without institutional preparedness evaluation. |
| 78 | Khan et al. | 2015 | Global | Scoping review | Included |  |
| 79 | Binder et al. | – | Global | Survey | Excluded | Descriptive survey without analytic assessment of preparedness performance. |
| 80 | Shah et al. | 2018 | Global | Book chapter | Included |  |
| 81 | Danjuma et al. | 2025 | Nigeria | Tool development | Excluded | Subnational assessment tool beyond national-system focus. |
| 82 | Rasanathan et al. | 2025 | Global | Policy analysis | Included |  |
| 83 | Kamga et al. | 2022 | Europe | Systematic review | Included |  |
| 84 | Haeberer et al. | 2021 | Europe | Application study | Excluded | Regional application of a tool rather than independent system analysis. |
| 85 | Miqdadi & Hamdan | 2024 | Palestine | Qualitative | Included |  |
| 86 | Asiedu-Berkoe et al. | 2022 | Ghana | Empirical | Included |  |
| 87 | Erondu et al. | – | Africa | Commentary | Excluded | Opinion piece lacking empirical methods. |
| 88 | Carbone & Thomas | 2018 | Global | Conceptual | Included |  |
| 89 | Hayes et al. | 2024 | Global | Tool study | Included |  |
| 90 | Souza et al. | 2025 | Brazil | Historical analysis | Included |  |
| 91 | Príncipe et al. | – | Global | Modelling paper | Excluded | Modelling framework not grounded in real-world system assessment. |
| 92 | Hill et al. | 2023 | Global | Technical paper | Excluded | Genomic surveillance focus unrelated to PHEPR governance. |
| 93 | Elhakim et al. | 2024 | EMR | Report | Excluded | Described training interventions without assessment of preparedness systems. |
| 94 | Meena et al. | 2024 | Global | Clinical review | Excluded | Disease-specific clinical preparedness focus, not system-level PHEPR. |
| 95 | Lyu et al. | 2025 | Multi-country | Comparative study | Excluded | Compared pandemic responses without analyzing preparedness institutions. |
| 96 | Daszak et al. | 2021 | Global | Essay | Excluded | Opinion piece without empirical or systematic methods. |
| 97 | Kostkova | 2018 | Global | Ethics paper | Excluded | Focused on data ethics rather than preparedness systems. |
| 98 | Khan et al. | 2020 | Global | Policy paper | Excluded | Addressed information systems only without evaluation of institutional preparedness. |
