# Supplementary material for: Building systems for preparedness: Global scoping studies on institutional governance and National Public Health Agencies
Source: PLOS Glob Public Health. 2026 Feb 12;6(2):e0005427. doi: 10.1371/journal.pgph.0005427 (PMC12900297; doi:10.1371/journal.pgph.0005427)
Supplement: S3 Table — This table provides structured data extraction for the 60 included studies, including study characteristics, key findings, limitations, policy implications, and analytical domains. (DOCX) [file pgph.0005427.s004.docx]

S3 Table. Detailed data extraction for studies included in the scoping review (n = 60). Data extractors: Dr. Sileshi Demelash Sasie and Dr. Fantu Mamo Argaw. Date of data extraction: April 2025. Search coverage period: March 2000 to April 2025.

| **ID** | **Authors & Year** | **Region** | **Study Type** | **Focus Area** | **Data Source** | **Key findings** | **Limitations** | **Policy implications** | **Domains** |
| --- | --- | --- | --- | --- | --- | --- | --- | --- | --- |
| 1 | Ostermann, 2023 | Europe | Policy commentary | Role of NPHIs post-COVID | Policy analysis | Reports that NPHIs became visible “system hubs” during COVID-19, particularly for data analytics, risk communication, and cross-sector coordination. Notes that post-crisis expansion of functions often outpaced formal mandate reforms, leaving responsibilities uneven across countries. Highlights that political shift can rapidly alter institutional space, affecting continuity of preparedness functions. | Commentary design; relies on European experience and illustrative examples rather than systematic multi-country measurement. | Legal codification of NPHI mandates and protected baseline financing are positioned as prerequisites for sustaining gains; clarifying subnational coordination roles is emphasized to prevent preparedness being trapped at central level. | Governance |
| 2 | Frieden & Koplan, 2010 | Global | Commentary | Strengthening NPHIs | Expert perspective | Describes NPHIs as national integrators that consolidate surveillance, laboratory coordination, workforce development, and outbreak support under one institutional umbrella. Frames fragmentation as a recurring barrier to coherent IHR-aligned preparedness. Emphasizes learning networks and institutional partnerships as mechanisms to accelerate capacity development. | Opinion-driven; does not provide empirical tests or stepwise implementation pathways for resource-constrained systems. | Promotes WHO-aligned institutional models, structured peer networks/twinning, and domestic financing strategies so NPHIs can operate beyond project cycles. | Governance, IHR |
| 3 | Herstein et al., 2021 | Global | Perspective | Future preparedness | Narrative review | Argues that preparedness should be treated as an adaptive system rather than a static plan, with governance that can adjust under uncertainty. Highlights the operational value of digital analytics, scenario-based planning, and continuous learning loops. Notes that siloed institutions and episodic funding patterns undermine readiness between crises. | Conceptual perspective; does not provide outcome evaluation or comparative testing of proposed approaches. | Recommends institutionalizing adaptive governance practices, investing in analytics capacity linked to decision processes, and funding recurring exercises and stress tests to maintain readiness. | Systems resilience |
| 4 | Myhre et al., 2022 | Global | Scoping review | NPHI landscape | Literature review | Maps global presence of NPHIs and shows wide heterogeneity in mandate scope, autonomy, and resource base. Identifies definitional inconsistency as a barrier to benchmarking and cross-country learning. Notes that formal establishment does not reliably predict operational maturity or integration with emergency systems. | Dependent on available published sources; under-captures informal arrangements and unpublished institutional realities. | Calls for typologies, standardized indicators, and regional peer review approaches to support comparability and improvement planning. | Governance |
| 5 | Sasie et al., 2024 | Ethiopia | Mixed methods | Information sharing | Interviews, documents | Documents fragmented information flow across institutions, with limited interoperability, inconsistent data-sharing practices, and unclear stewardship. Notes that coordination weaknesses are not only technical but also procedural, including absence of harmonized protocols and accountability for information exchange. Describes how these gaps constrain timely situational awareness during emergencies. | Primarily national-level focus; limited measurement of downstream effects on decision quality or response outcomes. | Proposes formal data-sharing frameworks, enforceable MOUs, and interoperable platforms so surveillance, laboratories, and coordination units operate in a single information environment. | Coordination |
| 6 | Abou-Taleb et al., 2024 | EMR | Regional review | NPHIs | Expert mapping | Describes uneven NPHI development across the region, from mature institutes acting as IHR focal points to nascent entities with limited authority. Highlights reliance on external support and variability in domestic financing, affecting sustainability. Notes weak regional coordination despite ongoing technical support initiatives. | Descriptive regional mapping; limited depth on within-country performance variation and few outcome metrics. | Emphasizes legislation to formalize mandates, domestic financing streams to reduce donor dependence, and strengthening regional technical networks for peer learning. | Governance |
| 7 | Khatri et al., 2023 | LMICs | Qualitative synthesis | Preparedness gaps | Multi-study synthesis | Synthesizes evidence that surveillance, laboratory reach, and surge workforce readiness remain common weak points in fragile systems. Identifies that community-facing adaptations can partially compensate but are limited without clear authority and resourcing. Notes dependence on external aid as a structural constraint that shapes preparedness continuity. | Heterogeneous evidence base and contexts; limited ability to attribute findings to specific institutional models. | Prioritizes core capacities, clearer institutional leadership and coordination, and establishment of routine monitoring baselines to support sustained system strengthening. | Capacity |
| 8 | Chiossi et al., 2021 | Global | Scoping review | Preparedness tools | Tool mapping | Catalogues tools used for preparedness assessment and training, showing increasing institutionalization of exercises and self-assessment practices. Notes weak validation and poor comparability across tools and settings. Highlights that many tools originate in high-income contexts with unclear transferability to LMIC environments. | Pre-COVID timeframe; English-language scope; limited evidence on real-world uptake and performance impacts. | Recommends standardization and validation across contexts and making tools accessible for adaptation, including LMIC-relevant guidance. | Evaluation |
| 9 | Calonge et al., 2020 | USA | Commentary | Evidence use | NASEM report | Argues that preparedness decisions often occur without structured mechanisms to incorporate evidence in real time. Emphasizes the operational importance of documenting decision rationales, evidence sources, and learning from interventions. Positions evidence systems as part of preparedness infrastructure, not an academic add-on. | Commentary format; limited examples of implemented models and no comparative evaluation. | Encourages evidence logs, rapid review functions, and tighter alignment between research agendas and decision-maker needs inside emergency operations. | Decision making |
| 10 | Sasie et al., 2025a | Ethiopia | Validation study | PHEM tool | Survey validation | Reports development of a six-domain assessment tool spanning governance, coordination, workforce, logistics, surveillance, and financing, with strong internal reliability. Positions the tool as a structured way to identify gaps consistently across administrative levels. Highlights its potential to shift assessment from ad hoc judgement to repeatable measurement. | Validation limited to selected settings; limited external validation and benchmarking across contexts. | Recommends institutionalizing tool use through national and regional structures and aligning it with IHR monitoring and evaluation processes for routine application. | Evaluation |
| 11 | Savoia et al., 2009 | USA | Review | PHSR | Literature review | Finds preparedness evidence was limited in breadth and often disconnected from implementation realities. Notes that translation of research into actionable institutional reforms was inconsistent. Highlights the need for systems research approaches that reflect how preparedness functions operate in practice. | Pre-COVID and largely US-centered evidence base; limited LMIC representation. | Calls for expanding preparedness systems research globally and strengthening implementation-focused designs that inform policy and organizational change. | Research |
| 12 | Lamberti-Castronuovo, 2022 | Global | Review | PHC preparedness | Evidence synthesis | Concludes PHC is frequently treated as peripheral to emergency planning, despite being central to continuity of care and early response. Notes that PHC integration is often ad hoc, with weak financing and unclear operational roles during crises. Highlights gaps in preparedness-linked IPC, supply continuity, and facility readiness support. | Review-level evidence; limited country case depth and variable definitions of PHC preparedness. | Recommends explicit PHC preparedness planning, PHC-specific budget lines, and strengthening IPC and stockpiling capacity at frontline facilities. | PHC |
| 13 | English et al., 2024 | Global | Methods paper | Operational readiness | Framework design | Proposes a readiness architecture with minimum viable capabilities across governance, information, logistics, workforce, and learning systems. Emphasizes routine drills, decision logs, and maturity cues to monitor readiness in peacetime. Frames readiness as operational performance, not only policy presence. | Early-stage framework; limited LMIC piloting and limited cost guidance. | Encourages adoption of readiness checklists, piloting in diverse settings, and attaching measurable KPIs that can be tracked across cycles. | Operations |
| 14 | Yang et al., 2022 | Global | Bibliometric | PHEM research trends | Database mapping | Maps thematic clusters in PHEM literature and shows rapid growth during COVID-era publishing. Identifies uneven emphasis on implementation science and limited integration between digital tools research and decision quality. Notes cross-disciplinary collaboration gaps in published outputs. | Descriptive mapping; database and language bias possible; does not assess effectiveness. | Suggests prioritizing applied research funding, shared taxonomies, and incentives for cross-disciplinary teams to strengthen practice-relevant evidence. | Knowledge |
| 15 | Rubinelli et al., 2022 | Global | WHO framework | Infodemic management | Framework | Defines competency areas for infodemic management spanning monitoring, analysis, intervention design, and evaluation, embedded within RCCE structures. Emphasizes social listening and feedback loops as routine functions, not crisis-only activities. Provides a structured basis for workforce development and role definition. | Limited empirical linkage to improved outcomes; assessment tools and benchmarking not standardized. | Supports embedding competencies into HR systems, developing certification/assessment rubrics, and integrating infodemic SOPs within PHEOC communication functions. | Communication |
| 16 | Sasie et al., 2025b | Ethiopia | Scoping review | Evaluation frameworks | Literature synthesis | Synthesizes frameworks into an eight-domain model of institutional maturity for PHEM, linking governance, leadership, financing, and learning systems. Identifies that LMIC operational evidence is limited relative to frameworks, creating gaps between theory and implementation. Positions maturity models as useful for structured planning and monitoring. | Evidence heterogeneity; limited LMIC empirical applications and underrepresentation of African validation cases. | Recommends applying and validating the framework using national data and aligning domains with WHO and Africa CDC benchmark systems. | Evaluation |
| 17 | Karo et al., 2018 | Multi-country | Evaluation / perspective | EWARS interoperability | Field / program evidence | Reports that interoperable EWARS can improve alert timeliness and consistency when data standards and thresholds are harmonized. Highlights the governance conditions needed for interoperability, including stewardship arrangements and formal agreements. Notes integration with HMIS as feasible but dependent on institutional alignment. | KPI reporting inconsistent across settings; adoption and sustainability vary by context. | Recommends defining EWARS KPIs, formal MOUs for data sharing, and harmonization with national digital health strategies and routine surveillance. | Surveillance |
| 18 | Chen et al., 2022 | Global | Empirical | Genomic surveillance | Global dataset | Documents rapid scale-up of sequencing and associated systems, while highlighting major regional disparities. Notes persistent weaknesses in metadata completeness and integration of genomics into operational dashboards. Positions bioinformatics capacity as a limiting step for sustained use. | Dynamic datasets; LMIC underrepresentation and evolving infrastructure complicate comparability. | Highlights need for durable financing, LIMS and metadata standards, integration with surveillance decision platforms, and workforce development in bioinformatics. | Laboratories |
| 19 | Brito et al., 2022 | Global | Empirical | Genomics inequity | Data analysis | Quantifies inequities in throughput and turnaround time that limit timely variant detection, with infrastructure concentrated in HICs. Notes structural barriers to equitable access, including funding concentration and technical capacity gaps. Highlights the role of hubs and shared pipelines in addressing constraints. | Attribution and causality limits; data rapidly evolve and reflect shifting capacity. | Emphasizes regional hubs, pooled financing, equity KPIs, and investment in technology transfer and mentorship as pathways to reduce disparities. | Equity |
| 20 | Gurley et al., 2021 | Global | Policy analysis | Essential services | Multi-country synthesis | Reports that essential health services are vulnerable during epidemics when governance does not define responsibilities between outbreak control and routine service delivery. Identifies the need for monitoring service disruptions and structured catch-up strategies. Notes that policy levers exist but are inconsistently institutionalized. | Limited quantitative evaluation and high context variability across health systems. | Recommends institutionalizing EHS continuity plans with triggers, monitoring indicators for disruptions, and financing mechanisms that enable flexible staffing and catch-up campaigns. | Service continuity |
| 21 | Hung et al., 2022 | Asia | Delphi framework | Health-EDRM | Consensus study | Articulates an all-hazards, multi-sector EDRM architecture emphasizing governance, community interfaces, and risk management functions. Identifies priority research and implementation gaps in financing, interoperability, and community resilience. Positions translation of research into SOPs as a persistent weakness. | Agenda-setting emphasis; limited piloting and limited cost detail. | Calls for cross-sector pilots, evaluation of scalability and cost-effectiveness, and embedding resulting guidance in national SOP repositories. | All-hazards |
| 22 | De Foo et al., 2022 | Global | Systematic review | PHSM | Evidence synthesis | Finds PHSM effectiveness depends on context and layered implementation, including trust, risk communication, and enabling supports. Notes that activation and de-escalation benefit from explicit thresholds and real-time monitoring. Highlights variability in effectiveness and equity impacts across settings. | Evidence heterogeneity and confounding; limited equity-stratified analyses in parts of the literature. | Recommends trigger-based PHSM design with built-in equity monitoring, dashboard-supported decision-making, and documented de-escalation criteria. | PHSM |
| 23 | Derese et al., 2025 | Ethiopia | Cross-sectional | Facility readiness | Survey | Reports moderate health-center preparedness scores with notable gaps in training, logistics, and simulation drills. Identifies supervision and coordination as weak operational supports, affecting readiness consistency. Presents facility-level preparedness as uneven even within an urban setting. | Self-reported measures; urban-only sample and disease-specific focus limit generalization. | Recommends continuous training and drills, stronger supervision systems, and functional surge financing pathways to support facility readiness. | PHC |
| 24 | Wright et al., 2024 | Global | Practice synthesis | Supply chains | Case analysis | Describes supply-chain resilience strategies including diversified procurement, stockpiling, and inventory visibility tools. Highlights surge-ready contracting and regulatory flexibility as operational enablers. Notes that data transparency supports fairer and faster allocation decisions during shortages. | Illustrative and often HIC-leaning cases; LMIC evidence less represented. | Recommends visibility platforms, pre-negotiated surge contracts, regional stockpiles, and routine stress tests using operational KPIs (stockouts, fill rates, turnaround times). | Logistics |
| 25 | Meyer et al., 2020 | Global | Framework | Resilience | Checklist | Presents a resilience checklist across governance, surveillance, workforce, and logistics domains, synthesizing lessons from outbreaks and disasters. Emphasizes embedding resilience in planning cycles rather than treating it as a separate agenda. Positions checklist use as a way to make readiness expectations explicit. | No field validation; limited guidance on measurement and scoring in diverse contexts. | Recommends piloting and adapting in LMIC settings, attaching indicators to checklist elements, and integrating into routine preparedness assessments. | Governance |
| 26 | Durski et al., 2020 | Global | Policy perspective | System reform | Perspective | Argues that crises can accelerate institutional reforms in governance, financing, and workforce organization. Highlights Ebola and COVID as catalysts for reconfiguring preparedness within broader health system strengthening. Notes the risk of reforms being temporary if not institutionalized post-crisis. | Conceptual framing; limited detailed case evidence or evaluation of reform impacts. | Recommends linking outbreak reviews to funded reform plans and embedding preparedness priorities within UHC and health system strengthening agendas. | Policy |
| 27 | Zhang et al., 2023 | Global | Narrative review | Systems improvement | Narrative | Describes how investments in surveillance, diagnostics, and countermeasures can produce spillover benefits for routine health systems. Highlights cross-sector collaboration as part of sustained infectious disease control. Frames system improvement as both a preparedness and routine health objective. | Limited empirical validation; narrative synthesis without outcome evaluation. | Recommends aligning vertical outbreak investments with routine system strengthening and ensuring coordination across programs and sectors. | Integration |
| 28 | Haldane et al., 2021 | Global | Comparative study | COVID resilience | Multi-country | Identifies governance clarity, trust, community engagement, and PHSM adaptability as recurring resilience enablers across countries. Notes inequities in financing and service continuity as important constraints on response effectiveness. Highlights variation in how systems learned and adapted during COVID. | Retrospective comparative design; heterogeneous contexts and data sources limit direct comparability. | Recommends resilience dashboards, documentation of good practices, and explicit equity safeguards integrated into preparedness planning. | Governance |
| 29 | Berkessa et al., 2025 | Ethiopia | System evaluation | Sentinel surveillance | WHO evaluation tool | Reports high completeness in sentinel reporting but persistent challenges in timeliness and feedback loops. Notes underuse of subnational data analysis and incomplete linkage with IDSR processes. Highlights sustainability questions for post-COVID continuation. | Sentinel-site scope; limited qualitative insight from frontline users and limited sustainability analysis. | Recommends strengthening IDSR integration, automating reporting processes, building analytic capacity, and improving feedback loops between laboratories and facilities. | Surveillance |
| 30 | Mackenzie et al., 2014 | Global | Program report | GOARN | Descriptive report | Describes GOARN as an operational surge mechanism that depends on predefined activation protocols, partner rosters, and training arrangements. Highlights recurring constraints in sustained funding and surge readiness. Notes the importance of networks for rapid deployment across borders. | Descriptive and overlapping with related GOARN literature; limited deployment impact metrics. | Recommends stronger measurement of deployment outputs and impacts and more stable financing to sustain readiness between emergencies. | Surge |
| 31 | Cordes et al., 2017 | LMIC | Case/program report | EWARN | Field reports | Describes EWARN value for early detection in crisis contexts, highlighting its flexibility and timeliness. Notes recurring operational challenges, including denominator uncertainty and quality assurance gaps. Points to sustainability problems when systems are not integrated into routine surveillance. | Variable data quality across contexts; uncertain sustainability after emergency phase. | Recommends standard quality checks, integration into national HMIS/IDSR, and training surge surveillance officers to maintain system function. | Surveillance |
| 32 | Kluge et al., 2018 | Europe | Policy perspective | IHR integration | Perspective | Argues that IHR capacities are more sustainable when embedded in domestic laws, governance arrangements, and routine budgeting. Highlights the importance of empowered National Focal Points and clear authority for coordination. Frames IHR as a system-wide obligation rather than a compliance exercise. | Policy perspective without empirical testing or implementation evaluation. | Recommends institutional budgeting for IHR functions, stronger NFP authority, and monitoring compliance across subnational levels. | IHR |
| 33 | Nuzzo et al., 2019 | Global | Scoping review | Resilience | Literature review | Finds resilience literature broad but fragmented, with inconsistent definitions and weak operational metrics. Identifies recurring elements (governance, financing, trust, integration, adaptability) but limited tools to measure them. Notes limited LMIC examples in published work. | Predominantly descriptive evidence; limited comparative metrics and LMIC representation. | Recommends development and piloting of resilience metrics and linking resilience assessment to preparedness scorecards and monitoring systems. | Evaluation |
| 34 | Stehling-Ariza et al., 2017 | Global | Program report | Rapid response | CDC case | Describes design of a global rapid response roster emphasizing multidisciplinary deployability and training. Notes its operational contributions across multiple emergencies and the value of readiness systems to mobilize staff quickly. Highlights challenges in sustaining readiness and evaluating outcomes. | Limited outcome evaluation; sustainability and impact metrics not fully developed. | Recommends formal KPIs for surge deployment, regular roster refresh cycles, and expanded partnerships that strengthen LMIC surge capacity. | Workforce |
| 35 | Martínez et al., 2019 | USA | Framework | Capability standards | Standards review | Summarizes evolution of capability-based preparedness standards that define functions for incident management, PHEOCs, surveillance, and workforce readiness. Positions standards as tools for consistent planning and accountability across jurisdictions. Notes variation in implementation across agencies. | US policy context; transferability may require adaptation. | Suggests harmonizing standards and developing repositories that support broader adoption and alignment with IHR monitoring approaches. | Operations |
| 36 | Tahir et al., 2025 | Pakistan | Case study | Infodemic | Provincial program data | Describes provincial experience integrating infodemic management within preparedness and response structures. Highlights operational gaps in communication capacity and resource shortages that affect coherence. Notes structured rumor tracking and communication workflows as practical mechanisms. | Single setting; limited comparative evaluation and outcome measurement. | Recommends dedicated RCCE/infodemic functions within PHEOCs, funded staffing, and workforce training tied to operational roles. | Communication |
| 37 | Wilbroda et al., 2024 | Kenya | Field survey | Community readiness | Field survey | Reports low levels of individual and community preparedness knowledge and limited household resources to act on guidance. Highlights the role of CHWs as trusted channels for preparedness messaging and local readiness activities. Notes readiness as uneven within communities. | Single locality and small sample; limited generalizability. | Recommends community awareness programming, CHW strengthening, and local preparedness scorecards to track change over time. | Community |
| 38 | Horney et al., 2019 | USA | Practice analysis | Capabilities use | Survey | Shows that agencies interpret and apply preparedness capabilities unevenly, contributing to variation in planning and performance. Highlights that capability frameworks require clear guidance to be operationally meaningful. Notes the importance of capability-based planning as a structuring mechanism. | Survey-based and self-reported; primarily US context. | Recommends clearer capability definitions, standardized guidance for application, and mechanisms for sharing practice examples to reduce variability. | Capability |
| 39 | Lee et al., 2023 | Global | Scoping review | Infectious emergencies | Evidence synthesis | Synthesizes evidence emphasizing integrated surveillance, laboratory functions, and PHEOC operations, with rapid learning cycles as a key enabler. Highlights workforce flexibility and routine coordination structures as repeated themes. Notes gaps in evaluation of PHEOC performance and surge systems. | Limited to scoped period and sources; excludes some grey literature. | Recommends expanding evaluation evidence in LMIC contexts, with particular focus on PHEOCs, surge workforce, and learning systems. | Systems |
| 40 | Murthy et al., 2017 | USA | Retrospective analysis | National progress | Data review | Describes major gains in laboratory capacity, surveillance, and incident management over time, alongside persistent weaknesses in equity, workforce sustainability, and cross-jurisdiction coordination. Notes preparedness progress as uneven and dependent on sustained investment. Highlights the challenge of maintaining readiness between emergencies. | US-only scope; retrospective design and reliance on national reporting structures. | Recommends equity-sensitive indicators, sustained workforce investments, and improved mechanisms for coordination across jurisdictions and sectors. | Evaluation |
| 41 | Marron et al., 2025 | Ireland | Narrative review | National framework | Document synthesis | Synthesizes COVID and EU lessons to inform a national preparedness framework emphasizing governance, surveillance, and coordination. Highlights the role of cross-border collaboration and standardized processes within European structures. Notes the framework orientation toward system organization rather than single interventions. | Narrative synthesis; limited empirical validation of framework performance. | Recommends piloting, alignment with EU standards, and strengthening cross-border collaboration mechanisms. | Policy |
| 42 | Ongesa et al., 2025 | Nigeria | Case study | Urban crises | Project management analysis | Presents urban emergency response as a coordination and logistics problem that can benefit from structured project management tools. Highlights complexity of stakeholder coordination and bottlenecks in resource movement and task tracking. Positions project management as a practical operational layer for preparedness. | Single-case illustration; limited generalizability and limited outcome measures. | Recommends adapting project management approaches for EPR, training urban managers, and evaluating feasibility and cost-effectiveness. | Operations |
| 43 | Nelson et al., 2007 | USA | Conceptual framework | Defining PHEP | Framework | Defines core constructs that distinguish preparedness from response, emphasizing planning, training, surveillance, communication, and legal/ethical scaffolds. Highlights measurability and accountability as necessary for operationalizing preparedness. Notes the value of shared definitions for consistent evaluation and dialogue. | Conceptual, without empirical testing; does not specify function-level indicators. | Recommends translating constructs into measurable indicators and embedding definitions into SOPs, training, and evaluation designs. | Conceptual |
| 44 | Stoto et al., 2017 | Europe | Logic model | Cross-border preparedness | Framework | Provides a logic model linking inputs, processes, outputs, and outcomes for cross-border threat management. Clarifies institutional roles and pathways for information sharing across jurisdictions. Positions logic models as tools for planning and evaluation of joint preparedness. | Limited field validation at scale; operational metrics not fully specified. | Recommends testing through cross-border exercises and agreeing shared KPIs and reporting templates. | Evaluation |
| 45 | Chiang et al., 2020 | USA | Framework/application | RWA readiness | Tool guidance | Operationalizes readiness across organizational supports, staff willingness, and technical ability, offering a structured self-assessment approach. Emphasizes use for targeting training and HR policy gaps that affect deployability. Positions readiness measurement as an input to continuous improvement. | Outcome linkage limited; primarily internal or programmatic applications reported. | Recommends adopting self-assessment within QI cycles and evaluating association with deployment and response performance. | Workforce |
| 46 | Kennedy et al., 2021 | USA | Observational evaluation | Accreditation & PHEPR | Observational analysis | Reports association between accreditation and stronger preparedness-related processes, including documentation, quality improvement, and governance routines. Suggests accreditation may reinforce organizational discipline relevant to preparedness, while acknowledging non-causal interpretation. Highlights variation across agencies. | Observational design; selection bias possible; causality not established. | Recommends embedding preparedness standards in accreditation processes and evaluating impacts using longitudinal approaches. | Quality |
| 47 | Bedi et al., 2021 | India | Narrative synthesis | Modelling | Practice synthesis | Describes practical requirements for embedding modelling and forecasting into preparedness decision cycles, including data governance and analyst–decision-maker interfaces. Emphasizes communicating uncertainty and integrating models with surveillance workflows. Positions analytic capacity as an institutional function rather than a one-off product. | Narrative orientation; limited operational performance metrics or comparative cases. | Recommends establishing modelling cells within PHEOCs, defining protocols for model use, and implementing data-sharing agreements and audit trails. | Analytics |
| 48 | Fu et al., 2021 | China | System description | Radiological EPR | National system description | Describes specialized command structures, surveillance, clinical guidance, and expert networks for radiological hazards, including drills and stockpiles. Highlights interagency linkages and periodic readiness activities as routine functions. Positions rare hazards as needing integration with general emergency architectures. | Country-specific description; limited detail on integration with broader EPR systems. | Recommends cross-training teams, joint exercises, and aligning communication and clinical pathways within general preparedness structures. | All-hazards |
| 49 | Davis et al., 2021 | USA | Program analysis | Workforce readiness | CDC systems | Describes workforce deployability policies including cross-training, readiness dashboards, credentialing, and exercise cycles. Highlights leadership support and protected training time as operational determinants. Notes gaps in linking training participation to deployment performance indicators. | US-centric program focus; limited outcome metrics and external comparators. | Recommends institutionalizing roster health indicators, linking training to deployment outcomes, and sustaining incentives that protect surge capacity. | Workforce |
| 50 | Hao et al., 2024 | China | Case / metric development | Urban EPR evaluation | Index/scorecard | Develops and applies a city-level preparedness scorecard covering governance, resources, services, and risk management, revealing uneven capacities across subdistricts. Demonstrates how composite indicators can guide targeted investment decisions. Notes the operational value of sub-city granularity for planning. | Single-province pilot; weighting and indicator choice may be subjective; transferability uncertain. | Recommends standardizing indicators across cities, validating the tool, and linking results to budgeting and remediation planning. | Evaluation |
| 51 | Khan et al., 2018 | Canada | Framework | PHEP as resilience | Conceptual framework | Frames preparedness as resilience capability, emphasizing adaptability, learning, redundancy, and community engagement alongside technical capacities. Highlights that resilience characteristics require routine tracking and organizational commitment. Positions learning systems as central to sustaining readiness. | Framework-level guidance; limited operational metrics and implementation detail. | Recommends translating resilience attributes into measurable KPIs and embedding them in capability assessments and after-action review processes. | Resilience |
| 52 | Khan et al., 2015 | Canada | Scoping review + consultation | Evidence base | Literature review | Maps the evidence base and finds limited rigorous primary research, heavy HIC bias, and few comparative/effectiveness studies. Identifies priority domains where stronger designs are needed, including surveillance, risk communication, and coordination. Highlights the importance of co-produced research agendas. | Pre-COVID era evidence; heterogeneity and datedness relative to current system reforms. | Recommends investment in quasi-experimental and longitudinal studies and aligning research agendas with practice leaders’ operational questions. | Research |
| 53 | Shah et al., 2019 | USA | Conceptual / normative | Ethics | Policy discussion | Synthesizes ethical and legal considerations relevant to emergency decisions, including equity, proportionality, transparency, and privacy. Emphasizes that ethical principles require operational checkpoints to influence real decisions. Highlights documentation as part of accountability. | Non-empirical; limited implementation playbooks or evaluation evidence. | Recommends ethics checkpoints in decision cycles, staff training, and public transparency protocols during PHSM and allocation decisions. | Ethics |
| 54 | Rasanathan et al., 2025 | Global | Perspective | Governance | Governance analysis | Argues that governance design shapes preparedness performance, particularly statutory autonomy, budget authority, and accountability arrangements. Highlights leadership pipelines and policy coherence as determinants often overlooked in technical capacity discussions. Notes that agency structure influences credibility and action speed. | Perspective-based; lacks comparative metrics linking governance features to outcomes. | Recommends defining governance indicators, protecting technical autonomy through statutes, and routine public reporting to strengthen accountability. | Governance |
| 55 | Kamga et al., 2022 | Europe | Systematic review | Multisector | Evidence synthesis | Catalogues sectors involved in preparedness and identifies recurring weaknesses in legal clarity, data-sharing, and joint exercises. Highlights that multisector engagement exists but coordination mechanisms vary widely. Notes that whole-of-society approaches require institutional routines, not only policy statements. | Evidence dispersed and partly dependent on grey literature; uneven documentation across sectors. | Recommends formal MOUs, regular multi-agency exercises, and clearer legal bases for data exchange and emergency powers. | Coordination |
| 56 | Miqdadi & Hamdan, 2024 | Palestine | Qualitative study | Stakeholders | Interviews | Reports barriers including resource scarcity, fragmented authority, and limited training, alongside enabling roles for local leadership and NGO support. Highlights that subnational dynamics and community engagement shape what is feasible in practice. Presents context-specific needs for coordination and workforce development. | Single setting; qualitative scope limits generalizability and quantification. | Recommends co-designed local preparedness plans, subnational training and surge rosters, and basic performance monitoring to support incremental improvement. | Subnational |
| 57 | Asiedu-Berkoe et al., 2022 | Ghana | Assessment | National capacity | Situation analysis | Describes national and subnational EPR structures and reports moderate role definition with gaps in surveillance, laboratories, and workforce capacity. Highlights financing fragility and uneven capacity across districts. Notes sustainability concerns where partner support dominates. | Limited quantitative performance metrics and limited cross-district comparability. | Recommends investment in surveillance and laboratories, scaling FETP at all tiers, earmarked subnational grants tied to KPIs, and stabilizing domestic funding. | Capacity |
| 58 | Carbone & Thomas, 2018 | USA | Narrative / perspective | Evidence use | Perspective | Traces evolution toward evidence-informed preparedness and emphasizes institutional mechanisms for translating research into action. Highlights rapid reviews, decision support, and consistent evaluation as organizational functions. Notes persistent gaps between knowledge production and operational use. | Narrative perspective; limited adoption metrics and implementation evidence. | Recommends rapid evidence units in EOCs, evaluation plans for major interventions, and closer alignment between research funding and operational gaps. | Knowledge |
| 59 | Hayes et al., 2024 | Europe | Tool development | Workforce | Tool development | Develops a workforce competency self-assessment tool to identify preparedness-related strengths and gaps and support training and roster planning. Positions repeat use as a way to track improvement over time. Emphasizes practicality for organizational planning rather than academic measurement. | Early validation stage; benchmarking guidance and real-world adoption barriers not fully established. | Recommends multi-site piloting, clear scoring guidance, benchmarking approaches, and linking results to training budgets and HR planning decisions. | Workforce |
| 60 | Souza et al., 2025 | Brazil | Historical / policy analysis | PHEM evolution | Policy analysis | Chronicles how legal frameworks and governance shifts shaped national PHEM development, including incident management institutionalization and inter-federal coordination. Highlights reforms as iterative and influenced by past epidemics and disasters. Notes that institutional arrangements evolve with threat environments and political context. | Historical orientation; limited quantitative impact evaluation against contemporary performance metrics. | Recommends translating historical lessons into updated statutes and SOPs, formalizing inter-federal coordination compacts, and evaluating reforms using modern KPIs. | Governance |
