# Supplementary material for: Building systems for preparedness: Global scoping studies on institutional governance and National Public Health Agencies
Source: PLOS Glob Public Health. 2026 Feb 12;6(2):e0005427. doi: 10.1371/journal.pgph.0005427 (PMC12900297; doi:10.1371/journal.pgph.0005427)
Supplement: S4 Table — This table synthesizes characteristics, pertinent findings, identified gaps, and recommendations across all included studies. (DOCX) [file pgph.0005427.s005.docx]

S4 Table. Characteristics, key findings, limitations, and recommendations of studies included in the scoping review (n = 60)

| **Authors & Year** | **Continent / Region** | **Type of Study / Document** | **Areas of Focus** | **Pertinent Findings** | **Identified Gaps / Study Limitations** | **Recommendations** |
| --- | --- | --- | --- | --- | --- | --- |
| Ostermann, 2023[[1](#_ENREF_1)] | Europe (global relevance) | Commentary / Policy | Role of NPHIs post-COVID | NPHIs emerged as central actors during COVID-19, especially for analytics, risk communication, and coordination. Governments increasingly acknowledge their importance, yet mandates and autonomy remain inconsistent. Political volatility risks undermining newly gained capacities. | Limited to European experience; lacks comparative global analysis; does not include systematic data. | Legally codify NPHIs’ mandates; provide baseline financing; institutionalize their role within MoH structures and subnational systems. |
| Frieden et al., 2010[[2](#_ENREF_2)] | Global | Commentary | Strengthening national PH institutes | NPHIs are highlighted as crucial for implementing IHR and global health security. They consolidate surveillance, workforce development, and coordination functions. Peer learning and networking are emphasized as mechanisms for strengthening capacity. | Opinion-driven piece; lacks empirical validation or detailed LMIC pathways. | Develop WHO-aligned NPHI blueprint; create peer networks and twinning programs to support LMICs. |
| Herstein et al., 2021[[3](#_ENREF_3)] | Global | Perspective | Future of emergency preparedness | Emergency preparedness must be reframed around agility, digital analytics, and resilient governance. Scenario-based planning and adaptive systems are needed to avoid reactive responses. Current systems remain siloed and underfunded. | Conceptual; no piloting or quantitative evaluation included. | Roadmap national systems with simulations and stress tests; fund digital tools and workforce flexibility; embed adaptive governance into preparedness structures. |
| Myhre et al., 2022[[4](#_ENREF_4)] | Global | Scoping review | NPHI landscape | Provides a systematic mapping of NPHIs worldwide, showing heterogeneity in mandates, autonomy, and resources. Highlights the growth of NPHIs after the 2005 IHR, but also the lack of shared definitions that hinders cross-country learning. | Based on published sources; limited inclusion of grey literature or informal institutional arrangements. | Develop typologies and standardized indicators for NPHIs; promote regional peer review systems. |
| Sasie et al., 2024 **[**[5](#_ENREF_5)**]** | Africa (Ethiopia) | Mixed-methods empirical study | Inter-institutional information sharing and coordination during public health emergencies | Revealed fragmented information flow across ministries and agencies, absence of standardized protocols, and limited digital interoperability; identified weak data governance and accountability mechanisms. | Focused primarily on national-level actors; limited generalizability to local structures; did not assess information-sharing outcomes quantitatively. | Develop national data-sharing frameworks, enforce inter-agency memoranda of understanding (MOUs), and expand interoperable digital infrastructure to improve real-time coordination. |
| Abou-Taleb et al., 2024 [[6](#_ENREF_6)] | Eastern Mediterranean Region | Regional review | NPHIs in EMR | Documents uneven development of NPHIs in the region: some serve as IHR focal points, while others lack legal and financial foundations. Shows donor dependence and weak regional coordination, though WHO/EMRO has driven progress. | Descriptive mapping; limited country-level depth; few outcome measures. | Enact legislation to formalize NPHIs; secure domestic financing streams; strengthen regional technical networks. |
| Khatri et al., 2023[[7](#_ENREF_7)] | LMICs / Global South | Qualitative synthesis | Preparedness in weak health systems | Identifies systemic weaknesses in surveillance, coordination, and surge workforce capacity. Community-based practices such as health worker surveillance provided adaptive strategies, but overall authority remains fragmented and dependent on external aid. | Broad synthesis across heterogeneous data sources; lacks in-depth empirical testing. | Strengthen core capacities (surveillance, labs, workforce); clarify institutional leadership; build structured M&E baselines. |
| Chiossi et al., 2021[[8](#_ENREF_8)] | Europe-heavy (global) | Scoping review | PHEP tools & methods | Compile’s preparedness assessment and training tools from 2013–2019. Shows institutionalization of exercises and self-assessments, but comparability and validation remain weak. Tools are often designed in high-income contexts with unclear LMIC transferability. | Excludes COVID-19 lessons; limited to English-language publications. | Standardize and validate toolkits across multiple contexts; ensure open access for adaptation in LMICs. |
| Calonge et al., 2020[[9](#_ENREF_9)] | USA (global relevance) | Commentary (NASEM) | Evidence-based PHEPR | Argues for embedding evidence systematically into preparedness and response decisions. Stresses the importance of institutional mechanisms to ensure evidence-informed action during crises. | Short commentary; no empirical testing or pilot examples provided. | Require evidence logs in EOCs; align research agendas with operational decision-making needs. |
| Sasie et al., 2025a **[**[10](#_ENREF_10)**]** | Africa (Ethiopia) | Tool development and validation study | Development and validation of a national Public Health Emergency Management (PHEM) assessment tool | Produced a six-domain validated instrument covering governance, coordination, workforce, logistics, surveillance, and financing; Cronbach’s alpha = 0.89, indicating strong reliability. | Validation conducted in limited subnational settings; lacks external validation in other LMIC contexts. | Institutionalize tool use nationwide through EPHI and regional health bureaus; integrate into WHO IHR Monitoring & Evaluation Frameworks. |
| Savoia et al., 2009[[11](#_ENREF_11)] | USA | Review | Public health systems research in preparedness | Highlights public health systems research (PHSR) as a foundation for preparedness capacity-building. Shows that the evidence base was narrow, largely U.S.-centric, and poorly translated into practice. | Dated pre-COVID synthesis; excludes LMIC contexts. | Expand PHSR to global contexts; prioritize robust designs; strengthen translation of findings into policy. |
| Lamberti-Castronuovo et al., 2022[[12](#_ENREF_12)] | Global | Review | PHC disaster preparedness | Emphasizes PHC as essential for continuity of care during disasters. Shows that PHC is underutilized in pandemic preparedness, with integration into EPR often ad hoc and financing mechanisms weak. | Review only; limited depth on country-level case studies. | Integrate preparedness explicitly into PHC planning; allocate PHC-specific budgets; strengthen IPC and stockpiling at PHC facilities. |
| English et al., 2024[[13](#_ENREF_13)] | Global | Consensus framework / methods paper | Operational public health readiness | Proposes a practical readiness architecture spanning governance, information, logistics, workforce, and learning systems; defines maturity cues and minimum viable capabilities; emphasizes routine “peacetime” drills and decision logs; aligns readiness checks to incident command and after-action learning cycles. | Early-stage field validation; limited LMIC piloting; lacks cost guidance. | Adopt the readiness checklist nationally; pilot in diverse settings; attach KPIs (e.g., decision-to-action time, resource fill rates); budget for routine exercises. |
| Yang et al., 2022[[14](#_ENREF_14)] | Global (analysis led from China) | Bibliometric / knowledge mapping | Public health emergency management (PHEM) research landscape | Maps thematic clusters (surveillance, PHSM, risk comms, modeling, governance); shows surge in COVID-era publications but uneven focus on implementation science; identifies under-researched links between digital tools and decision quality; highlights limited cross-disciplinary collaboration. | Descriptive design; no outcomes; potential database/language bias. | Direct funding to practice-oriented topics (implementation, evaluation); incentivize cross-disciplinary teams; create shared taxonomies for PHEM keywords. |
| Rubinelli et al., 2022[[15](#_ENREF_15)] | Global | Framework (WHO) | Infodemic management competencies (RCCE) | Defines role-specific competencies across monitoring, analysis, intervention design, and evaluation; integrates with RCCE and risk governance structures; promotes continuous feedback loops and social listening; provides curriculum anchors for national training. | Limited empirical linkage to outcomes; assessment tools not standardized. | Embed competencies in HR frameworks; develop assessment rubrics and certification; integrate infodemic SOPs into PHEOC comms cells. |
| Sasie et al., 2025b **[**[16](#_ENREF_16)**]** | Africa (Ethiopia / global synthesis) | Scoping review (Arksey & O’Malley framework) | Frameworks for evaluating PHEM implementation and institutional maturity | Synthesized 72 sources to propose an 8-domain evaluation framework linking governance, leadership, financing, and learning systems; highlighted need for integrated institutional maturity models. | Limited empirical studies from LMICs; heterogeneity among included frameworks; underrepresentation of African case applications. | Apply and validate the proposed framework using national PHEM data; promote harmonization with WHO Benchmarks and Africa CDC NPHI frameworks. |
| Karo et al., 2018[[17](#_ENREF_17)] | Multi-country (WHO/EU focus) | Evaluation / perspective | EWARS interoperability | Shows interoperable EWARS improves timeliness and consistency of alerts; recommends standardized thresholds and data schemas; demonstrates feasible integration with HMIS; underscores need for formal MOUs to secure data sharing and clarify stewardship. | Sparse, comparable KPI reporting across countries; variable adoption. | Define national EWARS KPIs (TAT, PPV, completeness); sign data-sharing MOUs; harmonize with digital health strategies and routine surveillance. |
| Chen et al., 2022[[18](#_ENREF_18)] | Global | Empirical landscape analysis | Genomic surveillance | Documents rapid global scale-up of sequencing; reveals significant regional disparities and metadata quality issues; stresses integration of genomics with epidemiological dashboards; argues for durable post-emergency financing and bioinformatics capacity. | LMIC under-representation; evolving datasets; uneven metadata completeness. | Fund LMIC sequencing and LIMS; standardize metadata; link genomics to surveillance decision dashboards; support bioinformatics training. |
| Brito et al., 2022[[19](#_ENREF_19)] | Global | Empirical analysis | Genomic surveillance disparities | Quantifies inequities in throughput and turnaround time that constrain variant detection and response; identifies concentration of funding and infrastructure in HICs; proposes regional hubs and shared pipelines; highlights technology transfer and open protocols as accelerators. | Dynamic data; attribution challenges for causality. | Establish regional sequencing hubs; design pooled financing mechanisms; track equity KPIs (coverage, TAT); invest in tech transfer and mentorship. |
| Gurley et al., 2021[[20](#_ENREF_20)] | Global | Policy analysis / synthesis | Maintaining essential health services (EHS) during epidemics | Identifies policy levers to protect EHS alongside PHSM (prioritization matrices, alternate care pathways, telehealth); underscores governance clarity between EHS and outbreak control; shows importance of monitoring service disruptions and catch-up strategies. | Limited quantitative evaluation; context variability across health systems. | Institutionalize EHS continuity plans with triggers; track disruption indicators; fund catch-up campaigns and flexible staffing. |
| Hung et al., 2022[[21](#_ENREF_21)] | Asia (globally applicable) | Framework → research agenda | Health-EDRM | Articulates an all-hazards, multi-sector EDRM framework; prioritizes community interfaces and risk governance; sets a research agenda on financing, interoperability, and community resilience; emphasizes translation of research to SOPs. | Agenda-setting without large-scale pilots; limited cost/financing detail. | Fund cross-sector EDRM pilots; evaluate scalability and cost-effectiveness; embed results in national SOP repositories. |
| De Foo et al., 2022[[22](#_ENREF_22)] | Global | Comprehensive/systematic review | Public health & social measures (PHSM) | Synthesizes evidence for layered PHSM and local tailoring; highlights dependencies on trust, risk communication, and enabling services; advocates adaptive M&E with clear activation/de-activation thresholds; notes variable effectiveness across settings. | Heterogeneity and confounding limit precision; few equity-stratified analyses. | Implement trigger-based PHSM with built-in equity assessments; establish real-time monitoring dashboards; document de-escalation criteria. |
| Derese et al., 2025 **[**[23](#_ENREF_23)**]** | Africa (Ethiopia – Addis Ababa) | Cross-sectional facility-based study | Health-center-level preparedness for cholera outbreak response | Found moderate facility preparedness (62% score); identified critical shortages in workforce training, logistics, and simulation drills; supervision and coordination were suboptimal. | Self-reported data may introduce bias; limited to urban settings and single disease focus. | Strengthen continuous training and simulation exercises, improve supervision systems, and ensure surge financing mechanisms for outbreak response. |
| Wright et al., 2024[[24](#_ENREF_24)] | Global (HIC-leaning) | Case analysis / practice synthesis | Supply-chain resilience & countermeasures | Details strategies for diversified suppliers, regional stockpiles, and inventory visibility; emphasizes surge-ready contracts and regulatory flexibilities; links data transparency to allocation fairness and speed; proposes operational KPIs (fill rate, stockouts, TAT). | Illustrative cases; generalizability may vary; limited LMIC examples. | Invest in visibility platforms; pre-negotiate surge contracts; establish regional stockpiles; monitor SCM KPIs and conduct scenario stress tests. |
| Meyer et al., 2020[[25](#_ENREF_25)] | Global | Checklist / framework | Health system resilience | Proposes a resilience checklist across governance, surveillance, workforce, and logistics; highlights lessons from outbreaks and natural disasters; suggests institutionalizing resilience into planning cycles. | No empirical validation; checklist not piloted in LMICs. | Test and adapt checklist in LMICs; attach monitoring indicators; integrate into preparedness assessments. |
| Durski et al., 2020[[26](#_ENREF_26)] | Global | Policy / perspective | Using outbreaks to strengthen systems | Argues for using crises to accelerate reforms in governance, workforce, and financing; highlights Ebola and COVID lessons; proposes embedding outbreak response into health system strengthening agendas. | Conceptual framing; lacks detailed case evidence. | Fund health system reforms triggered by outbreak reviews; integrate EPR into UHC frameworks. |
| Zhang et al., 2023[[27](#_ENREF_27)] | Global | Policy synthesis | Infectious disease control systems improvement | Link’s disease control to system-wide improvements; shows how surveillance, diagnostics, and countermeasures have spillover benefits for routine health systems; highlights cross-sector collaborations. | Limited empirical validation; primarily narrative. | Leverage investments in outbreak control to strengthen routine health; align vertical and horizontal strategies. |
| Haldane et al., 2021[[28](#_ENREF_28)] | Global (28 countries) | Comparative study | Health system resilience in COVID-19 | Cross-country synthesis of COVID responses; identifies governance clarity, trust, community engagement, and PHSM adaptability as resilience enablers; highlights inequities in financing and service continuity. | Heterogeneous country contexts; retrospective design. | Develop resilience dashboards; document good practices; prioritize equity safeguards in preparedness planning. |
| Berkessa et al., 2025 **[**[29](#_ENREF_29)**]** | Africa (Ethiopia – national surveillance network) | System evaluation using WHO framework | Performance of ILI/SARI sentinel surveillance during COVID-19 (2021–2023) | Achieved >80% data completeness but low timeliness (64%); data analysis underused at subnational levels; weak feedback to reporting sites and incomplete IDSR integration. | Evaluation limited to sentinel sites; lacked qualitative insights from frontline staff; did not assess sustainability post-COVID. | Strengthen IDSR linkage and automated reporting, expand workforce training, and enhance feedback loops between laboratories and health facilities. |
| Mackenzie et al., 2014 [[30](#_ENREF_30)] | Global | Perspective / program note | GOARN | Complementary to Lancet article; elaborates on GOARN’s global deployments and networking; emphasizes challenges of sustained funding and surge readiness. | Overlaps with other GOARN papers; descriptive. | Consolidate GOARN evidence base; publish deployment impact metrics. |
| Cordes et al., 2017[[31](#_ENREF_31)] | LMIC emergencies | Case/program reports | EWARN in crises | Demonstrates utility of EWARN for early detection in emergencies; highlights timeliness and adaptability; shows challenges with denominator data and quality assurance. | Variable data quality; sustainability uncertain after emergencies. | Standardize quality checks; build EWARN into national HMIS; train surge surveillance officers. |
| Kluge et al., 2018[[32](#_ENREF_32)] | Europe (WHO) | Policy perspective | Embedding IHR into systems | Advocates embedding IHR requirements into routine systems; emphasizes financing and legal anchoring; calls for stronger NFP roles. | Policy perspective; lacks empirical examples. | Institutionalize IHR in budgeting; strengthen NFP authorities; monitor compliance at subnational level. |
| Nuzzo et al., 2019[[33](#_ENREF_33)] | Global | Scoping review | Health system resilience | Reviews evidence on resilience enablers: governance, financing, trust, integration, adaptability; concludes resilience literature is fragmented and lacks operational metrics. | Mostly descriptive; lacks LMIC examples. | Develop resilience metrics; pilot in LMIC contexts; tie resilience to preparedness scorecards. |
| Stehling-Ariza et al., 2017[[34](#_ENREF_34)] | Global / CDC | Case/program report | CDC Global Rapid Response Team | Describes establishment of GRRT; emphasizes multidisciplinary roster and rapid surge; outlines training model; shows value during Zika and Ebola responses. | Limited outcome evaluation; sustainability challenges. | Formalize surge KPIs; refresh rosters; expand partnerships with LMIC institutions. |
| Martínez et al., 2019[[35](#_ENREF_35)] | USA | Standards / framework | Preparedness capability standards | Outline’s evolution of U.S. capability standards; codifies incident management, PHEOC, surveillance, workforce; provides structured benchmarks for states. | Uptake uneven across jurisdictions; limited global applicability. | Harmonize standards; create global capability repository; integrate with IHR monitoring. |
| Tahir et al., 2025[[36](#_ENREF_36)] | South Asia (Pakistan) | Case / program report | Infodemic management and preparedness | Presents provincial experience in Khyber Pakhtunkhwa; shows integration of infodemic control with EPR; highlights institutional gaps in communication and resource shortages. | Case-limited; lacks comparative evaluation. | Build infodemic management units within PHEOCs; allocate funding; train workforce in RCCE. |
| Wilbroda et al., 2024[[37](#_ENREF_37)] | Africa (Kenya) | Field study / survey | Community-level preparedness | Survey in Kisumu shows low individual and community readiness for emergencies; highlights knowledge gaps and lack of resources; identifies importance of CHWs. | Limited to one setting; small sample size. | Strengthen community awareness programs; invest in CHWs; develop local preparedness scorecards. |
| Horney et al., 2019[[38](#_ENREF_38)] | USA | Practice analysis | Use of PHEP capabilities | Examines how agencies apply preparedness capabilities; shows wide variation in interpretation; emphasizes importance of capability-based planning. | Survey-based; mostly U.S. context. | Clarify capability definitions; standardize application; share best practices across jurisdictions. |
| Lee et al., 2023[[39](#_ENREF_39)] | Global | Scoping review | Infectious disease emergencies preparedness | Synthesizes recent evidence on PHEP; emphasizes integrated surveillance, labs, and PHEOCs; highlights rapid learning cycles and workforce flexibility. | Review scope limited to 2010s onward; excludes grey literature. | Expand scoping to LMIC data; prioritize evaluations of workforce surge and PHEOCs. |
| Murthy et al., 2017[[40](#_ENREF_40)] | USA | Retrospective national analysis | U.S. preparedness progress (2001–2016) | Shows major progress in labs, surveillance, and incident management post-9/11; highlights gaps in equity, workforce sustainability, and cross-jurisdictional coordination. | U.S.-focused; retrospective only. | Develop equity-sensitive indicators; sustain long-term workforce investments; enhance interstate coordination. |
| Marron et al., 2025[[41](#_ENREF_41)] | Europe (Ireland) | Narrative review | Health threats preparedness framework | Provides narrative evidence base for Irish preparedness framework; synthesizes lessons from COVID and EU reports; emphasizes governance, surveillance, and coordination. | Narrative only; limited empirical validation. | Pilot preparedness framework; integrate with EU standards; build cross-border collaboration. |
| Ongesa et al., 2025[[42](#_ENREF_42)] | Africa (Nigeria-led, regional relevance) | Project case / analysis | Urban health crises, emergency response | Applies project management to urban emergencies; highlights coordination complexity and logistics bottlenecks; proposes structured project management as a preparedness tool. | Single-case illustration; limited generalizability. | Adapt project management approaches for EPR; train urban managers; evaluate cost-effectiveness. |
| Nelson et al., 2007[[43](#_ENREF_43)] | USA (global relevance) | Conceptual framework | Defining public health emergency preparedness (PHEP) | Establishes a shared conceptual boundary between “preparedness” and “response,” proposing core constructs (planning, training, surveillance, communication, legal/ethical scaffolds) and stressing measurability and accountability; provides a baseline lexicon that later capability models drew upon, improving cross-agency dialogue and evaluation design. | Conceptual only; no empirical validation; function-level indicators unspecified. | Translate constructs into measurable indicators; pilot across jurisdictions; embed definitions in SOPs and training. |
| Stoto et al., 2017[[44](#_ENREF_44)] | WHO European Region | Framework / Logic model | Cross-border preparedness | Articulates a logic model to map inputs→processes→outputs→outcomes for cross-border threats; clarifies institutional roles (EU/EC, ECDC, national focal points) and pathways for information sharing; provides a scaffold to plan and evaluate joint preparedness across national boundaries. | Model not field-validated at scale; limited operational metrics. | Test the logic model through EU cross-border exercises; define shared KPIs and reporting templates. |
| Chiang et al., 2020[[45](#_ENREF_45)] | USA (transferable) | Framework / Application note | “Ready, Willing, Able” (RWA) readiness | Operationalizes agency readiness along three domains (organizational supports, staff willingness, technical ability); offers survey tools and guidance to target gaps; supports linking training and HR policies to deployability. | Limited outcome linkage; primarily internal CDC perspective. | Adopt RWA self-assessment; incorporate into QI and performance reviews; study association with deployment outcomes. |
| Kennedy et al., 2021[[46](#_ENREF_46)] | USA | Observational / program evaluation | Accreditation & PHEPR | Explores associations between public health accreditation and preparedness capability; suggests accreditation processes (QI, documentation, governance) align with stronger preparedness performance and continuous improvement cultures, though causality is not established. | Observational design; potential selection bias; limited generalizability. | Embed PHEPR standards into accreditation; evaluate causal impacts with longitudinal designs. |
| Bedi et al., 2021[[47](#_ENREF_47)] | India (global relevance) | Narrative / practice synthesis | Surveillance, modelling, forecasting | Summarizes practical requirements for modelling/forecasting within PHEPR: data governance, analyst–decision-maker interfaces, uncertainty communication, and integration into IM cycles; argues for dedicated analytic teams connected to surveillance and policy. | Narrative orientation; limited operational case metrics. | Stand up modelling cells in PHEOCs; define decision protocols for model use; build data-sharing agreements and audit trails. |
| Fu et al., 2021[[48](#_ENREF_48)] | China | System description | Nuclear/radiological emergency response | Describes legal/command structures, specialized surveillance, clinical guidance, and expert networks for radiological events; highlights periodic drills, stockpiles, and interagency linkages; offers a template to integrate rare but high-impact hazards into PHEOC structures. | Country-specific; integration with generic EPR not fully detailed. | Cross-train general EPR and radiological teams; run joint exercises; align communication protocols and clinical pathways. |
| Davis et al., 2021[[49](#_ENREF_49)] | USA | Program analysis | Workforce readiness (CDC) | Details policies for deployability: cross-training, readiness dashboards, credentialing, and exercise cycles; underscores leadership support and protected training time as determinants of surge capacity; identifies gaps in linking training to deployment performance. | US-centric; outcome metrics limited. | Institutionalize cross-training and roster health indicators; link training participation to deployment outcomes; maintain surge incentives. |
| Hao et al., 2024[[50](#_ENREF_50)] | China (Henan) | Case / metric development | Urban EPR capability evaluation | Proposes a resilience-oriented scorecard for city-level preparedness (governance, resources, services, risk management); demonstrates application in Henan; shows how composite indicators reveal uneven subdistrict performance, guiding targeted investments. | Single-province pilot; transferability uncertain; weighting choices subjective. | Standardize urban scorecards; validate across cities; tie to budget allocation and remediation plans. |
| Khan et al., 2018[[51](#_ENREF_51)] | Canada (global relevance) | Framework | PHEP as resilience | Introduces a resilience-focused framework for PHEP that emphasizes adaptability, learning, community engagement, and systems integration; encourages organizations to track resilience capabilities (e.g., redundancy, flexibility, reflective learning) beyond traditional checklists. | Framework-level guidance; lacks implementation detail and metrics. | Translate resilience attributes into KPIs; embed into capability assessments and after-action reviews. |
| Khan et al., 2015[[52](#_ENREF_52)] | Canada (global evidence) | Scoping review + consultation | Primary research in PHEP | Maps the pre-2015 evidence base, finding limited rigorous primary research, heavy HIC bias, and few comparative/effectiveness studies; highlights priority domains (surveillance, risk communication, coordination) where robust designs are needed. | Evidence heterogeneity; dated relative to COVID era. | Fund quasi-experimental and longitudinal PHEP studies; co-produce research agendas with practice leaders. |
| Shah et al., 2019[[53](#_ENREF_53)] | USA (general) | Conceptual / normative | Ethical & legal aspects of PHEPR | Synthesizes ethical/legal considerations (equity, proportionality, transparency, consent/privacy) and proposes integrating ethics checkpoints into preparedness planning, PHSM decision-making, and resource allocation; argues ethics must be operationalized, not ad hoc. | Non-empirical; lacks implementation playbooks. | Add ethics gates and documentation to EPR decision cycles; train staff on ethical frameworks; public transparency protocols. |
| Rasanathan et al., 2025[[54](#_ENREF_54)] | Global | Perspective / governance analysis | Governance of national public health agencies | Argues governance design (board vs ministerial control, statutory autonomy, budget authority) materially shapes preparedness performance; highlights policy coherence, leadership pipelines, and accountability mechanisms as under-addressed determinants of EPR quality. | Perspective-based; cross-country comparative metrics absent. | Define and test governance indicators; reform statutes to protect technical autonomy; publish annual governance scorecards. |
| Kamga et al., 2022[[55](#_ENREF_55)] | Europe (EU/EEA) | Systematic review | Multisector involvement in PHEPR | Catalogues sectors engaged in European PHEPR (health, interior, transport, education, private sector); maps coordination mechanisms and gaps in cross-sector exercises, data-sharing, and legal clarity; underscores whole-of-society requirements. | Evidence dispersed across grey literature; sectoral roles unevenly documented. | Formalize cross-sector MOUs; run multi-agency exercises; clarify legal bases for data exchange and emergency powers. |
| Miqdadi & Hamdan, 2024[[56](#_ENREF_56)] | Palestine | Qualitative exploratory | Stakeholder perspectives on PHEP | Surfaces practical barriers (resource scarcity, fragmented authority, limited training) and enablers (local leadership, NGO support); illuminates subnational dynamics and community engagement realities; provides context-specific priorities for capacity building. | Single setting; qualitative scope limits generalizability. | Co-design local preparedness plans with stakeholders; build subnational training and surge rosters; establish basic performance monitoring. |
| Asiedu-Berkoe et al., 2022[[57](#_ENREF_57)] | Ghana | Assessment / situation analysis | National & subnational EPR capacity | Describes national and regional/district structures; finds moderate autonomy, defined roles, but gaps in surveillance, labs, and workforce; funding mixes domestic and partners with sustainability concerns; reveals uneven subnational coverage and deployment. | Limited quantitative performance metrics; cross-district comparability unclear. | Invest in surveillance and labs; scale FETP (all tiers); earmark subnational grants tied to KPIs; stabilize domestic funding. |
| Carbone & Thomas, 2018[[58](#_ENREF_58)] | USA (generalizable) | Narrative / perspective | Science as basis for PHEPR practice | Traces the evolution from experience-based to evidence-based PHEPR; argues for institutional mechanisms to translate research into practice (evidence syntheses, rapid reviews, decision support); calls for consistent evaluation of interventions. | Narrative; lacks concrete adoption metrics. | Create rapid evidence units in EOCs; mandate evaluation plans for major interventions; align research funding with operational gaps. |
| Hayes et al., 2024[[59](#_ENREF_59)] | Europe-led (transferable) | Tool development | Workforce self-assessment for PHEP | Develops a practical self-assessment tool to map workforce competencies and gaps across preparedness functions; supports planning of training, surge rosters, and career pathways; suitable for repeated use to track improvement. | Tool requires field validation and benchmarking; adoption barriers unknown. | Pilot in multiple PHAs; add scoring guidance and benchmarking; link outputs to training budgets and HR plans. |
| Souza et al., 2025[[60](#_ENREF_60)] | Brazil | Historical / policy analysis | PHEM evolution | Chronicles Brazil’s PHEM development: legal frameworks, governance shifts, incident management institutionalization, and inter-federal coordination; highlights lessons from past epidemics and disasters for contemporary reform. | Historical orientation; limited quantitative impact evaluation. | Synthesize lessons into current statutes and SOPs; formalize inter-federal EPR compacts; evaluate outcomes against contemporary KPIs. |
