# Supplementary material for: Building systems for preparedness: Global scoping studies on institutional governance and National Public Health Agencies
Source: PLOS Glob Public Health. 2026 Feb 12;6(2):e0005427. doi: 10.1371/journal.pgph.0005427 (PMC12900297; doi:10.1371/journal.pgph.0005427)
Supplement: Attachment — Submitted filename: Response to Reviewers.docx [file pgph.0005427.s007.docx]

Cover Letter – Revised Manuscript Submission

Manuscript ID: PGPH-D-25-03097

Title: Building Systems for Preparedness: Global Scoping Studies on Institutional Governance and National Public Health Agencies

Journal: PLOS Global Public Health

Dear Dr. Janin,

Thank you for the opportunity to revise and resubmit our manuscript. We appreciate the constructive feedback from the editors and the two reviewers. We have carefully addressed all comments and journal requirements, and the manuscript has been substantially revised.

Key revisions include the following:

- The inclusion period for the scoping review has been harmonized across all sections to a single timeframe, with a clear methodological justification now provided.
- All Data Availability requirements have been fully met. We have added a complete inventory of included and excluded studies with explicit reasons for exclusion, a structured data extraction table with all variables required to replicate the review, and clear statements on risk of bias considerations and handling of missing data.
- The Results and Discussion sections have been extensively restructured to remove redundancy, strengthen focus on empirical findings, and sharpen the central analytical thesis.
- Terminology has been standardized throughout the manuscript, with clarification on the functional equivalence of National Public Health Agencies (NPHA) and National Public Health Institutes (NPHI).
- Ethiopia-specific discussion has been balanced to maintain an appropriately global perspective.
- All figures and tables have been reformatted in line with journal specifications, and supplementary files have been correctly designated.

Detailed, point-by-point responses to each reviewer and editor comment are provided in the accompanying “Response to Reviewers” document. A tracked-changes version and a clean revised manuscript are also included as requested.

We believe the revisions have significantly strengthened the manuscript and have fully addressed the concerns raised. Thank you for your consideration.

Sincerely,

Dr. Sileshi Demelash Sasie

Ethiopian Public Health Institute

Addis Ababa, Ethiopia

Corresponding Author, On behalf of all authors

**Response to Journal Requirements**

**Comment 1:** Please confirm the parameters for the literature included in your scoping review. The abstract states literature between 2007 and 2025 were included. In the methods, the manuscript mentioned including literature from 2000 to 2025 in the “Eligibility” section and literature from 2015 to 2025 in the “Information Sources and Search Strategy” section.

**Response:** We thank the editors for noting this inconsistency. The discrepancy resulted from an earlier draft of the protocol and was unintentionally retained in different sections of the manuscript. The correct and final inclusion period for the review is 2007–2025.

**Revision:** All relevant sections of the manuscript, including the Abstract, Eligibility Criteria, and Information Sources and Search Strategy, have been revised to consistently state the inclusion period as 2007–2025.

**Comment 2:** As required by our policy on Data Availability, please ensure your manuscript or supplementary information includes detailed tables of included and excluded studies, data extraction tables, risk of bias assessments where applicable, and an explanation of how missing data were handled.

**Response:**
We have fully complied with the PLOS Global Public Health Data Availability policy. The following supplementary materials have been prepared and uploaded as Supporting Information:

- **S1 Table:** Inventory of all studies assessed at full text, indicating included and excluded studies with specific reasons for exclusion
- **S2 Table:** Detailed search strategies across databases
- **S3 Table:** Data extraction framework
- **S4 Table:** Characteristics, key findings, limitations, and recommendations of included studies

These materials provide full transparency of the review process and enable replication of all analyses. The manuscript text and Data Availability Statement have been revised to reference these files appropriately. Missing data handling procedures have also been clarified in the Methods section.

**Comment 3: Financial Disclosure**

**Response:** The online Financial Disclosure statement has been updated to read:
“The authors received no specific funding for this work.”

**Comment 4: Competing Interests**

**Response:** The Competing Interests statement has been updated to read:
“The authors have declared that no competing interests exist.”

**Comment 5: Figure formatting**

**Response:** All main figures have been converted to .tif format in accordance with journal guidelines, and each file is under the required 10MB size limit.

**Comment 6: Figure legend**

**Response:** A separate legend for Figure 1 has been added within the manuscript.

**Comment 7: Tables formatting**

**Response:** All main tables have been incorporated directly into the manuscript as editable, cell-based objects. Only supplementary tables remain uploaded as separate Supporting Information files, as required.

**Comment 8: Supplementary file type**

**Response:** The file previously uploaded as “Supplementary materials.docx” has been re-uploaded correctly as Supporting Information with appropriate legends and citations within the manuscript.

**Comment 9: Reviewer-suggested citations**

**Response:**
All suggested publications were carefully reviewed. Relevant citations have been incorporated where appropriate. No non-relevant references were added.

**Response to Reviewer #1**

**Comment 1:** Length and redundancy

**Response:** We appreciate this feedback. The Results section has been streamlined to focus on empirical patterns, while the Discussion has been refocused on interpretation, implications, and contribution. Redundant text and repeated citations have been removed throughout, resulting in a clearer and more concise manuscript.

**Comment 2:** Sharpen the central analytical thesis

While the core insight is clear, it is diffused across sections. The reviewer recommends explicitly articulating the central argument early in the Introduction and reiterating it at the start of the Discussion.

**Response:** We agree that the manuscript benefits from a more explicit and consistently presented central argument. To address this point, we have revised both the Introduction and the Discussion.

A clear statement has now been added to the Introduction to frame the review from the outset. This statement makes explicit that the review examines preparedness effectiveness as a function of institutional integration, governance autonomy, sustainable financing, and subnational implementation, rather than as a matter of technical capacity alone.

**Revision**: Although substantial literature describes individual components of preparedness, less attention has been paid to how these components interact as part of an institutional system. This review therefore advances a central argument: preparedness effectiveness depends less on the mere presence of institutions than on their integration within legally mandated, autonomous, and sustainably financed governance systems with empowered subnational implementation. **(Introduction section, page 5, line 131-136)**

**Revision:** The Discussion now opens by restating this same argument, providing a coherent anchor for the interpretation that follows. All subsequent sections of the Discussion have been aligned with this thesis, so that governance, financing, workforce, subnational systems, and cross-cutting enablers are analyzed as interconnected components of a single systems-level explanation. These changes ensure that the manuscript presents a focused and unified analytical narrative, directly addressing the reviewer’s recommendation to sharpen and foreground the central argument.

**Comment 3:** There are minor inconsistencies in the reported publication date ranges across sections (i.e., 2007–2025 in the Abstract, 2000–2025 in the Methods). Harmonize and clearly justify a single inclusion period to avoid confusion for readers and reviewers.

**Response:** We thank the reviewer for identifying this inconsistency. The variation in date ranges resulted from an earlier draft revision that was not fully aligned across sections. We have now harmonized the inclusion period throughout the manuscript to a single, consistent timeframe of 2000–2025, and added a brief justification for this range in the Methods section.

**Revision:** A scoping review of literature published between 2000 and 2025 was conducted following the Arksey and O’Malley framework and reported according to PRISMA-ScR standards. **(Abstract section, page 2, line 33-35)**

**Comment 4:** Multiple related terms (PHEP, PHEPR, EPR) are used interchangeably. Standardize terminology and define alternatives once.

**Response:** We agree that the interchangeable use of related terms created ambiguity. In response, we have standardized terminology across the manuscript. The term public health emergency preparedness and response (PHEPR) is now used consistently as the primary descriptor, with alternative terms defined once and not repeated thereafter.

- All instances of PHEP and EPR have been replaced with the unified term PHEPR throughout the manuscript.

**Revision:** For consistency, this manuscript uses the term public health emergency preparedness and response (PHEPR) to refer to the full range of capacities required to prevent, detect, prepare for, and respond to public health emergencies. Related terms such as PHEP and EPR appear in the literature but are not used interchangeably in this review. **(Background section, page 5-6, line 136-140)**

- All tables, figures, and headings were reviewed and updated to reflect the standardized terminology.
- The search strategy description in the Methods now clarifies those multiple related terms were used for retrieval but are reported using the unified term PHEPR.

**Comment 5**: Several paragraphs are lengthy and could be split for clarity.

**Response**: We appreciate this observation. The manuscript has been carefully revised to improve readability and flow. Long paragraphs throughout the Introduction, Results, and Discussion sections have been divided into shorter, more focused units. Transitions between paragraphs were strengthened to ensure clearer progression of ideas and to enhance accessibility for readers.
**Revision:** Paragraph structure has been revised across the manuscript, with particular attention to improving clarity and coherence in the Introduction, Results, and Discussion sections.

**Comment 6:** Table 1 (seven-theme synthesis) could be strengthened to better highlight policy implications.
**Response:** We thank the reviewer for this helpful suggestion. Table 1 has been substantially revised to improve clarity and practical relevance. A new column has been added that explicitly summarizes policy implications and operational relevance for each of the seven themes. Descriptions within the table have been streamlined to better differentiate empirical findings from their implications, and terminology has been aligned with the main text.

**Revision:** Table 1 has been restructured to include an additional column titled “Policy and System Implications”, with clearer thematic summaries and refined wording.

**Comment:** Minor typographical and formatting issues should be addressed during revision.
**Response:** We appreciate this comment. The manuscript has undergone a comprehensive language and formatting review. Typographical errors, citation inconsistencies, and formatting issues have been corrected throughout the document. Reference formatting has been standardized, headings have been aligned with journal style, and minor grammatical issues have been addressed.
**Revision:** Editorial corrections have been implemented across all sections of the manuscript, including references, tables, and figure legends.

**Reviewer #2**

Comment 1: Please comment and clarify the difference between NPHA and NPHI (if any in the context of the paper) and specify in the results whether the findings apply to one or both.

**Response:** We appreciate this important question. In the international literature, the terms National Public Health Agency (NPHA) and National Public Health Institute (NPHI) are often used to describe national institutions with comparable mandates for surveillance, laboratory coordination, and public health emergency preparedness and response. The distinction between these terms is primarily administrative and contextual rather than functional. Because the included studies used both terms to describe institutions performing similar roles, the review treats them as equivalent for the purposes of synthesis.

**Revisions:** For consistency, this review uses the term National Public Health Institute (NPHI) as an overarching designation for national-level public health bodies responsible for surveillance, laboratory coordination, and public health emergency preparedness and response. In the global literature, the terms National Public Health Agency (NPHA) and National Public Health Institute (NPHI) are often used interchangeably to describe institutions with similar mandates. Where included studies used either term, both were considered functionally equivalent for the purposes of this synthesis. **(Background section, page 5, line 97-103)**

**Revision:** The search strategy incorporated both terms, including “National Public Health Institute,” “National Public Health Agency,” and related descriptors. Studies referring to either institutional model were eligible, provided they addressed national entities with responsibility for preparedness and response functions. **(Method section, page 10, line 219-222)**

**Comment 2:** The case of Ethiopia is well highlighted, but the discussion is more in-depth than for other countries. Please clarify whether there is an objective reason for examining the data relating to Ethiopia in greater detail; otherwise, please reduce the level of detail.

**Response:** We thank the reviewer for this observation. Ethiopia was initially described in greater detail because several empirical studies from Ethiopia were included in the review and provided concrete illustrations of broader thematic findings. However, we agree that the result presentation and discussion should maintain a balanced global focus consistent with the objectives of a scoping review.

**Revisions made:** The result and discussion section has been revised to substantially reduce Ethiopia-specific detail. References to Ethiopia are now presented only as brief illustrative examples alongside evidence from other countries. Country-level interpretations have been minimized to ensure that the Discussion remains proportionate and globally oriented.

**Comment 3:** Introduce practical and operational recommendations in the short term, or at least prioritize the proposed implications.

**Response:** Thank you for the opportunity to revise this section. The Policy Implications text has been carefully updated in line with the guidance provided. Revisions focused on improving clarity, coherence, and professional tone, and on presenting the implications in a structured, actionable manner. The section now more clearly emphasizes governance clarity, sustainable financing, workforce institutionalization, subnational empowerment, digital interoperability, routine learning processes, and alignment of international assistance with national systems. All changes have been incorporated while preserving a results-driven and policy-relevant focus. These revisions are reflected in the manuscript under the Policy Implications section **(pages 31–33, lines 691–718).**

**Comment 4:** The abstract and results refer to the period 2007–2025; however, the eligibility criteria refer to 2000–2025. Correct if this is a typo, or explain if there is a real difference in the period.

**Response:** We thank the reviewer for identifying this inconsistency. The reference to 2007–2025 was an unintended error from an earlier draft.

All sections of the manuscript have been harmonized to reflect a single, consistent inclusion period of 2000–2025. The Abstract, Methods, and Results now uniformly report this timeframe, and the Methods section clearly justifies the selected period.

**Revisions:**

A scoping review of literature published between 2000 and 2025 was conducted using PubMed, Scopus, Embase, Web of Science, CINAHL, and Google Scholar, following Arksey and O’Malley methodology and PRISMA-ScR reporting standards. **(Abstract section, line 33-35)**

Studies published from January 2000 through April 2025 were eligible for inclusion. The year 2000 was selected to align with the modern phase of public health emergency preparedness, characterized by IHR implementation and the expanding role of National Public Health Institutes as core national mechanisms for preparedness and response [20, 26-28]. This timeframe reflects the modern era of institutionalized public health emergency preparedness and response. **(Method section, page 9, line 202-207)**

**Comment 5**: In Strengths and limitations there is the following sentence: “Grey literature inclusion helped mitigate this gap but also introduced variability in methodological rigor.” Expand further, considering the potential weight of including grey literature in the review.

**Response:** We appreciate this insightful comment. Inclusion of grey literature was a deliberate methodological decision designed to capture policy reports, technical guidance, and operational evaluations that are not commonly available in peer-reviewed journals.

**Revisions:** The study also has limitations. As a scoping review, it maps the breadth of available evidence but does not formally assess methodological quality or evaluate the effectiveness of specific interventions. The findings rely on accessible published sources, which may underrepresent unpublished program experiences and rapidly evolving practices. Inclusion of grey literature strengthened the review by capturing national assessments, policy documents, and operational reports that are essential to understanding real-world preparedness, particularly in low- and middle-income settings. However, grey literature varies in methodological transparency and analytical rigor, introducing heterogeneity into the evidence base and requiring cautious interpretation. Differences in terminology and reporting across studies further limited direct comparability, and contextual variations between countries restrict the generalizability of some observations. **(Strength and limitation section, page 32, line 679-689)**
